# Aryl hydrocarbon receptor signaling modulates antiviral immune responses: ligand metabolism rather than chemical source is the stronger predictor of outcome

**DOI:** 10.1038/s41598-018-20197-4

**Published:** 2018-01-29

**Authors:** Lisbeth A. Boule, Catherine G. Burke, Guang-Bi Jin, B. Paige Lawrence

**Affiliations:** 1 Department of Environmental Medicine, University of Rochester School of Medicine and Dentistry, Rochester, NY USA; 2 Department of Microbiology and Immunology, University of Rochester School of Medicine and Dentistry, Rochester, NY USA; 3 Present Address: CBR International, Boulder, CO USA; 4 Present Address: Department of Preventative Medicine, School of Medicine, Yaniban University, Yanji City, Jilin Provence China

## Abstract

The aryl hydrocarbon receptor (AHR) offers a compelling target to modulate the immune system. AHR agonists alter adaptive immune responses, but the consequences differ across studies. We report here the comparison of four agents representing different sources of AHR ligands in mice infected with influenza A virus (IAV): TCDD, prototype exogenous AHR agonist; PCB126, pollutant with documented human exposure; ITE, novel pharmaceutical; and FICZ, degradation product of tryptophan. All four compounds diminished virus-specific IgM levels and increased the proportion of regulatory T cells. TCDD, PCB126 and ITE, but not FICZ, reduced virus-specific IgG levels and CD8^+^ T cell responses. Similarly, ITE, PCB126, and TCDD reduced Th1 and Tfh cells, whereas FICZ increased their frequency. In *Cyp1a1*-deficient mice, all compounds, including FICZ, reduced the response to IAV. Conditional *Ahr* knockout mice revealed that all four compounds require AHR within hematopoietic cells. Thus, differences in the immune response to IAV likely reflect variances in quality, magnitude, and duration of AHR signaling. This indicates that binding affinity and metabolism may be stronger predictors of immune effects than a compound’s source of origin, and that harnessing AHR will require finding a balance between dampening immune-mediated pathologies and maintaining sufficient host defenses against infection.

## Introduction

There is considerable evidence that signaling through the aryl hydrocarbon receptor (AHR) alters the course of adaptive immune responses in a manner that can be protective or detrimental. Adaptive immune responses underlie host protection from pathogens, but when improperly controlled they contribute to numerous diseases. The AHR’s remarkable capacity to modulate T cell responses has been demonstrated in autoimmune diseases^1–5^, allergic inflammation^6,7^, and inflammatory bowel diseases^8–10^. Yet, these reports also suggest that different AHR ligands may bias adaptive immune responses in opposite directions, and that exposure to the same ligand can worsen or improve pathology in different disease models^1,2,11^. While these issues remain to be resolved, the ability of the AHR to modulate T cell differentiation and T cell-dependent immune responses has generated enthusiasm about targeting therapeutic agents at the AHR in order to modulate the progression of a large spectrum of immune-mediated diseases^12,13^.

Yet, there is another aspect of AHR immunobiology that has direct bearing on the potential success of new strategies to use AHR ligands as treatment modalities: the impact on host responses to infection. Several reports demonstrate the importance of AHR in sensing microbes, including pathogenic and commensal bacteria, mycobacteria, and fungi^14–17^. Epidemiological studies show strong correlations between exposure to anthropogenically-derived AHR ligands from the environment and increased incidence and severity of respiratory infections, most notably viral infections^18,19^. These observations have been extended with animal studies, showing that AHR modulates cell-mediated and humoral immune responses to infection, and subsequently disease outcome^20^. A limitation of current information about AHR effects on adaptive immune responses during infection is that much of this evidence stems from studies conducted when AHR is activated using the high affinity binding environmental contaminant 2,3,7,8-tetrachlorodibenzo-*p*-dioxin (TCDD), which is resistant to metabolism. This raises questions about whether other compounds that bind AHR will similarly dampen key host protective adaptive immune responses to infection.

We report here a side-by-side comparison of the *in vivo* consequences of treatment with four different agonists on the adaptive immune response to infection with influenza A virus (IAV). To represent AHR binding compounds from different sources, we used 2,3,7,8-tetrachlorodibenzo-*p*-dioxin (TCDD), 3,3′,4,4′,5-pentachlorobiphenyl-126 (PCB126), 2-(1*H*-Indol-3-ylcarbonyl)-4-thiazolecarboxylic acid methyl ester (ITE), and 6-formylindolo(3,2-b)carbazole (FICZ). TCDD is the prototype and best characterized AHR ligand^21,22^. PCB126 is an abundant environmental contaminant with documented human exposure, yet its effects on the immune system remain understudied^23,24^. ITE represents pharmaceutical agents because of its potent AHR agonist activity *in vitro* and *in vivo*^25–27^, FICZ is a degradation product of tryptophan, and represents a naturally derived AHR ligand^28–31^. Infection with IAV elicits a vigorous adaptive immune response that involves virus-specific CD8^+^ cytotoxic T lymphocytes (CTL), conventional and regulatory CD4^+^ T cells, and virus-specific antibodies^32,33^. The overall magnitude of the adaptive response to IAV generally predicts the outcome following infection^32,34,35^. Using these four compounds, we compared CD8^+^ T cell, CD4^+^ T cell, and antibody responses to mild acute primary IAV infection. Additionally, we determined whether all of the observed effects require AHR in the immune system, and whether dampening ligand metabolism affected differences among the immunomodulatory effects of these compounds. By comparing the consequences of AHR activation by different types of compounds on the adaptive immune response to IAV infection, we extend our knowledge of ligand-specific, AHR-mediated effects on host responses to infection.

## Results

### Naturally-derived and anthropogenic AHR ligands elicit distinct effects on adaptive responses to acute primary influenza virus infection

**Figure 1:**
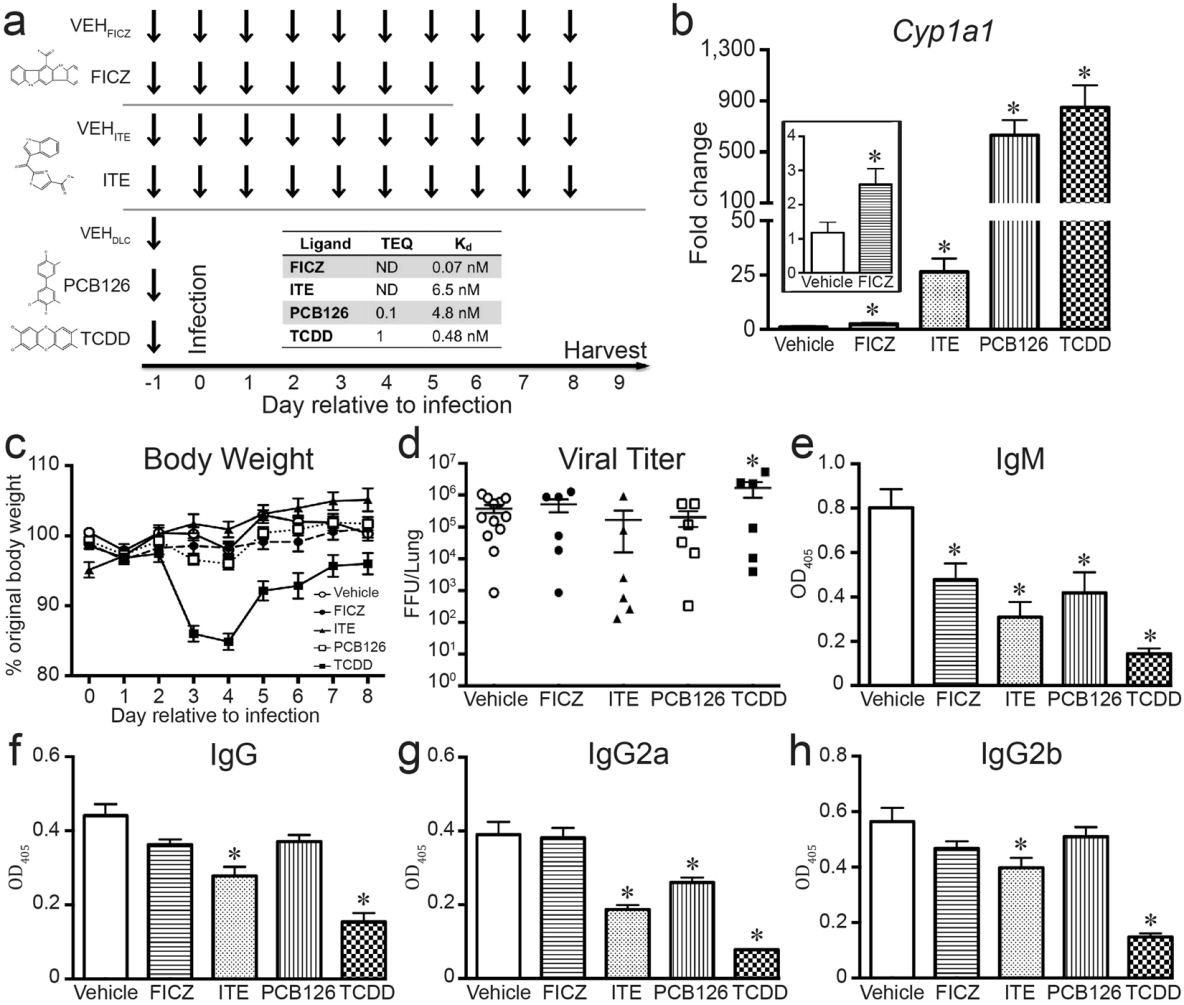
*In vivo* administration activates AHR. (**a**) Dosing strategy: arrows depict when female C57Bl/6 mice were treated with each compound. The indicated times are relative to intranasal (i.n.) infection with IAV, which is denoted as day 0. TCDD (10 μg/kg BW) and PCB126 (100 μg/kg BW) were administered orally once, one day before infection. FICZ (100 μg/kg BW daily) was also administered by gavage, whereas ITE (10 mg/kg BW daily) was given intraperitoneally (i.p.). Structures for each compound are shown to the left of the dosing strategy (www.chemspider.com). Control mice received the appropriate vehicle following the same treatment route and dosing schedule: VEH_FICZ_, VEH_ITE_, VEH_DLC_. The response of all vehicle treatment groups to infection was not different; therefore, a single representative vehicle group is shown in all figures. (**b**) RNA was isolated and RT-qPCR was performed to measure *Cyp1a1* levels. The graph depicts the mean expression of *Cyp1a1* levels in liver. The inset graph shows an enlargement of data comparing FICZ and vehicle treated mice. (**c**) The graph depicts the percent body weight change relative to the day prior to infection for mice in all treatment groups. (**d**) The pulmonary viral burden was measured 2 days after infection. The viral FFU per lung was determined by incubating lung homogenates on MDCK cells. Each symbol represents FFU/lung from a different mouse, and the horizontal line denotes the mean FFU for each treatment group. (**e**–**h**) Anti-influenza virus antibody ELISAs were performed using a dilution series of serum. The graphs show the mean level of circulating virus-specific (**e**) IgM (**f)** IgG, (**g**) IgG2a and (**h**) IgG2b in each group at the same serum dilution (1:6400). 5–8 mice were used per treatment group, and an * indicates a p value ≤ 0.05 as compared to appropriate vehicle control. All data are presented as the mean ± SEM. All experiments have been independently repeated at least once with similar results.

To directly compare the effects of these four compounds on the adaptive immune response to IAV, mice were randomly assigned to a group, dosed with each compound (or vehicle control), and infected as a group with the same viral inoculum (Fig. 1a). We selected a dose and route of exposure to each chemical based on prior reports, facilitating integration with findings in other model systems^2,25,27^. Briefly, the frequency of administration is based on current information regarding *in vivo* metabolism and elimination: FICZ is rapidly cleared, whereas PCB126 and TCDD are slowly to poorly eliminated, respectively^11,22,26^. The *in vivo* absorption, metabolism, distribution, and excretion rates of ITE are undetermined. Based on chemical structure, it is predicted to be more rapidly metabolized than TCDD or PCB126^25,27^; thus, dosing was daily. As a way of establishing *in vivo* activation of the AHR, we confirmed that administration of all 4 compounds significantly increased *Cyp1a1* expression in the liver (Fig. 1b). The induction of *Cyp1a1* in mice treated with FICZ was lower in magnitude relative to mice treated with ITE, PCB126, or TCDD (a 2.5-fold versus ≥ 25-fold increase over vehicle; Fig. 1b, inset). Previous reports showed that TCDD increases morbidity, and sometimes mortality, following IAV infection^36–39^. Therefore, we used a strain and dose of virus that causes a mild infection, in order to compare adaptive immune responses across the groups. With the virus inoculation used, only mice treated with TCDD exhibited severe weight loss (Fig. 1c), and none of the mice in any group died (data not shown). Yet, mice in all groups had similar lung viral burdens (Fig. 1d).

Primary infection with IAV stimulates production of virus-specific IgM and IgG antibodies, which are especially critical for viral clearance, and are important contributors to immunological memory, and host protection upon re-infection^40^. B cells secrete IgM independently of CD4^+^ T cell help, whereas switching to and secretion of IgGs requires T cells^41,42^. We measured circulating T cell-independent and T cell-dependent virus-specific immunoglobulin levels in infected mice. Treatment with all AHR ligands substantially reduced anti-IAV IgM levels in the blood (Fig. 1e). Virus-specific IgG and IgG2b levels were significantly reduced in ITE and TCDD groups (Fig. 1f,h), while circulating levels of virus-specific IgG2a were significantly reduced in mice treated with ITE, PCB126, and TCDD (Fig. 1g). In contrast, IgGs were unaffected in mice given FICZ (Fig. 1f–h). Thus, there are differences among these compounds in their effects on the relative amplitude of *Cyp1a1* induction, morbidity, and their specific impact on T-dependent (IgGs) versus T-independent (IgM) anti-viral antibody responses.

**Figure 2:**
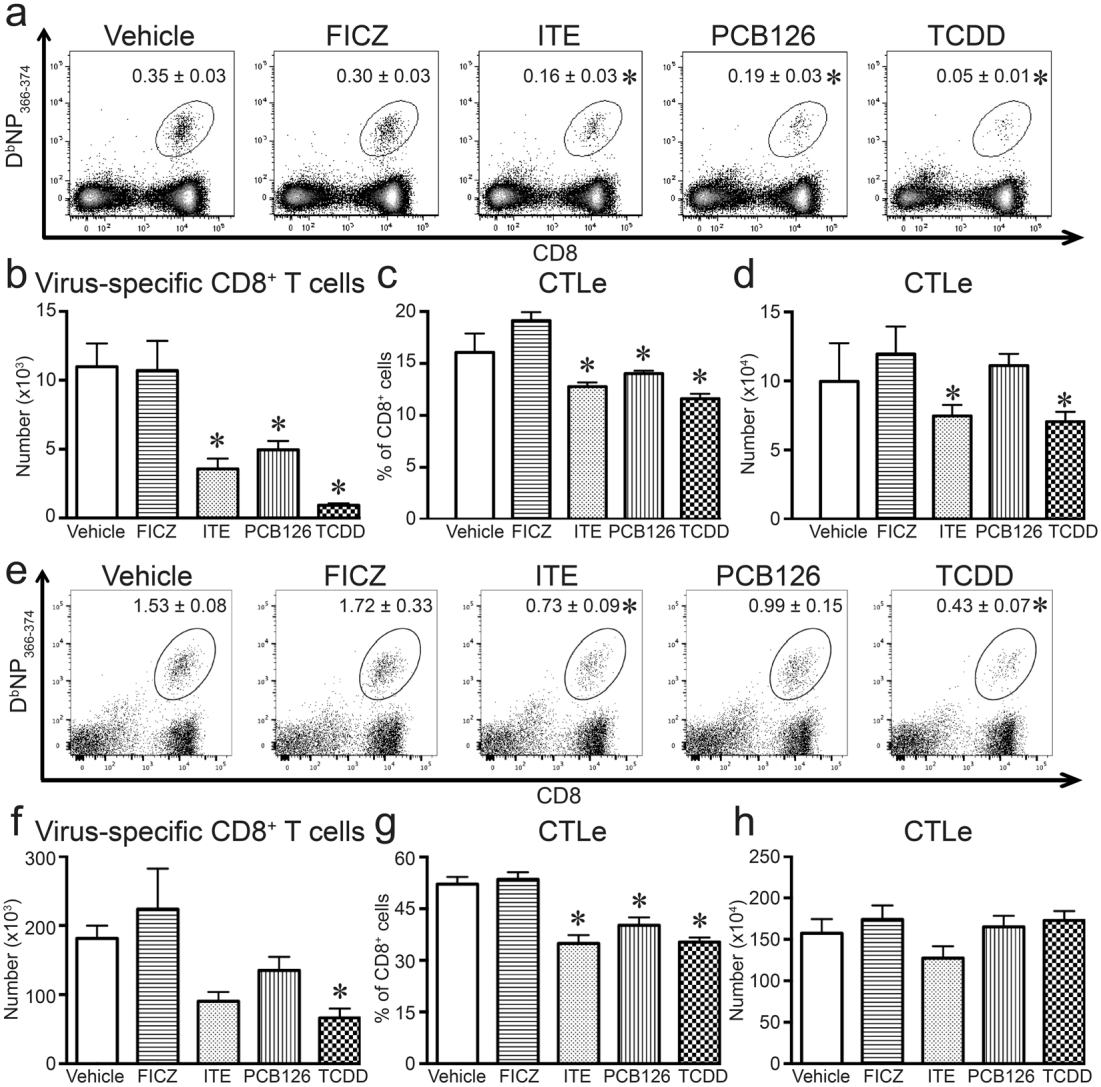
Virus-specific and cytotoxic CD8^+^ T cells are reduced by some AHR ligands. Mice were treated and infected as in Fig. 1. On day 9 post infection, MLN and lung derived immune cells were collected from the mice and stained for flow cytometry. (**a**) Representative dot plots depict D^b^NP_366–375_^+^CD8^+^ T cells in the MLN. The number on each plot is the mean percentage of CD8^+^ T cells labeled with D^b^NP_366–375_. (**b**) The graph shows the average number of D^b^NP_366–375_^+^CD8^+^ T cells in the MLN. (**c**,**d**) The bar graphs depict the (**c**) percentage and (**d**) number of cytotoxic effector cytotoxic T lymphocytes (CTLe) in the MLN, defined as CD44^hi^CD62L^lo^CD8^+^ T cells. (**e**) Representative dot plots depict D^b^NP_366–375_^+^CD8^+^ T cells in the lung. The number on each plot is the mean percentage of CD8^+^ T cells labeled with D^b^NP_366–375_. (**f**) The graph shows the average number of D^b^NP_366–375_^+^CD8^+^ T cells in the lung. (**g**,**h**) The bar graphs depict the (**g**) percentage and (**h**) number of cytotoxic CTLe in the lung. 5–8 mice per treatment group were used, and data are shown as the mean ± SEM. An * indicates p ≤ 0.05 compared to vehicle, and all experiments have been independently repeated at least one time with similar results.

Acute primary IAV infection also elicits a robust response from T cells, which play key roles in the antibody and cell-mediated immune responses to infection^32,33^. Following IAV infection, the responding CD8^+^ and CD4^+^ T cells proliferate and differentiate, reaching their peak magnitude 8–10 days later^43,44^. We measured the clonal expansion of CD8^+^ T cells that recognize an immunodominant peptide from the viral nucleoprotein (NP_366–372_), using fluorescently tagged tetrameric complexes of peptide-loaded MHC class I molecules (D^b^NP_366–374_). Treatment with ITE, PCB126, and TCDD reduced the percentage and number of virus-specific CD8^+^ T cells in the lung-draining mediastinal lymph nodes (MLN; Fig. 2a,b). ITE, PCB126, and TCDD also reduced the percentage and number of D^b^NP_366–374_^+^CD8^+^ T cells in the infected lung (Fig. 2e,f), although the effects of PCB126 were not statistically significant. In contrast, FICZ did not significantly change the percentage or number of virus-specific CD8^+^ T cells in the MLN (Fig. 2a,b) or infected lung (Fig. 2e,f). Given that NP-specific CD8^+^ T cells represent a subset of all CD8^+^ T cells that recognize and respond to IAV, we also compared the effects of these four compounds on the entire pool of CD8^+^ effector cytotoxic T lymphocytes (CD44^hi^CD62L^lo^CD8^+^ T cells; CTLe) in the MLN and lung. Extending prior reports that TCDD decreases CD8^+^ T cell differentiation, administration of ITE and PCB126 reduced the frequency of CTLe in the MLN (Fig. 2c,d) and lung (Fig. 2g,h). In contrast, daily treatment with FICZ did not significantly change the percentage or number of CTLe compared to infected mice given the vehicle control (Fig. 2c,d,g,h).

**Figure 3:**
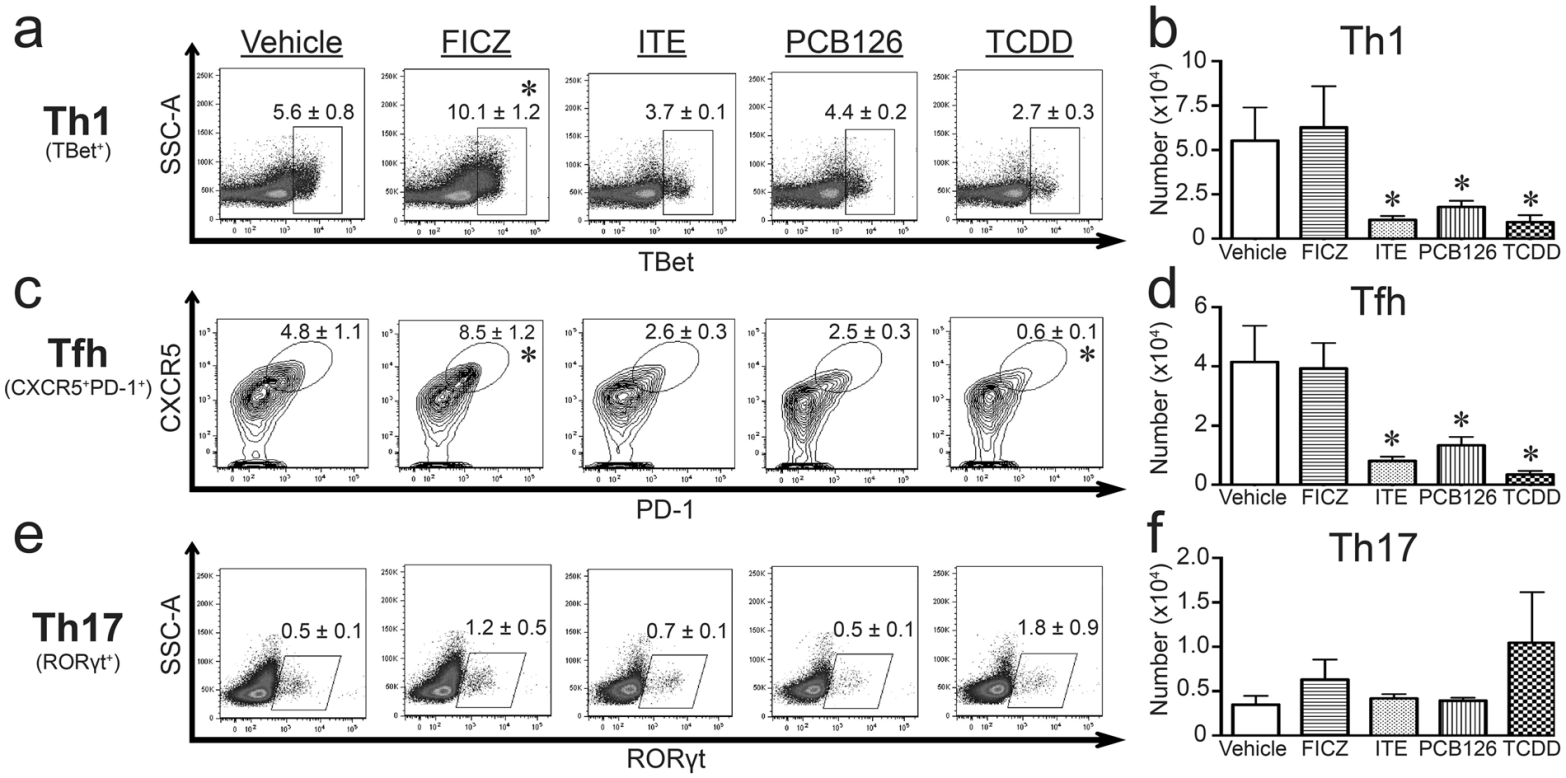
AHR ligand treatment alters conventional CD4^+^ T cell responses during infection in a ligand-specific manner. Mice were exposed and infected as described in Fig. 1. MLNs were harvested 9 days after infection, and cells were stained for flow cytometry. (**a**,**c**,**e**) Representative plots with mean percentage (± SEM) of CD4^+^ T cells that are (**a**) Th1 cells (TBet^+^CD4^+^, gated on CD4^+^ cells), (**c**) Tfh cells (CD44^hi^CXCR5^+^PD-1^+^CD4^+^, gated on CD44^hi^CD4^+^ cells), and (**e**) Th17 cells (RORγt^+^CD4^+^; gated on CD4^+^ cells). (**b**,**d**,**f**) The graphs show number (± SEM) of (**b**) Th1 cells, (**d**) Tfh cells, and (**f**) Th17 cells. 5–8 mice were used per group, and an * indicates a p value ≤ 0.05 compared to vehicle control. All experiments have been independently repeated at least once and yielded similar results.

In other model systems, AHR modulates the differentiation of CD4^+^ T cell subsets^12,13^. Therefore, we compared the consequences of *in vivo* exposure to these four agents on conventional and regulatory CD4^+^ T cells in the context of primary IAV infection. There were no differences in size of the total pool of CD4^+^ T cells in MLNs among infected mice treated with any of the four compounds and vehicle controls (data not shown). Yet, when distinct subsets were identified, differences are evident. The two most prevalent conventional CD4^+^ T cell subsets generated during acute IAV infection are Th1 cells and T follicular helper (Tfh) cells^44^. Exposure to FICZ doubled the percentage of Th1 and Tfh cells in IAV infected mice (Fig. 3a,c), although it did not significantly change the number of Th1 and Tfh cells (Fig. 3b,d). In contrast to FICZ, treatment with ITE, PCB126, and TCDD significantly (p ≤ 0.05) reduced the number of Th1 and Tfh cells (Fig. 3b,d). Given that AHR ligands can affect CD4^+^ T cell differentiation into Th17 cells^1,2,45^, we also examined Th17 cells in mice infected with IAV. Unlike Th1 and Tfh cells, none of these compounds altered the percentage or number of Th17 cells during acute primary IAV infection (Fig. 3e,f).

**Figure 4:**
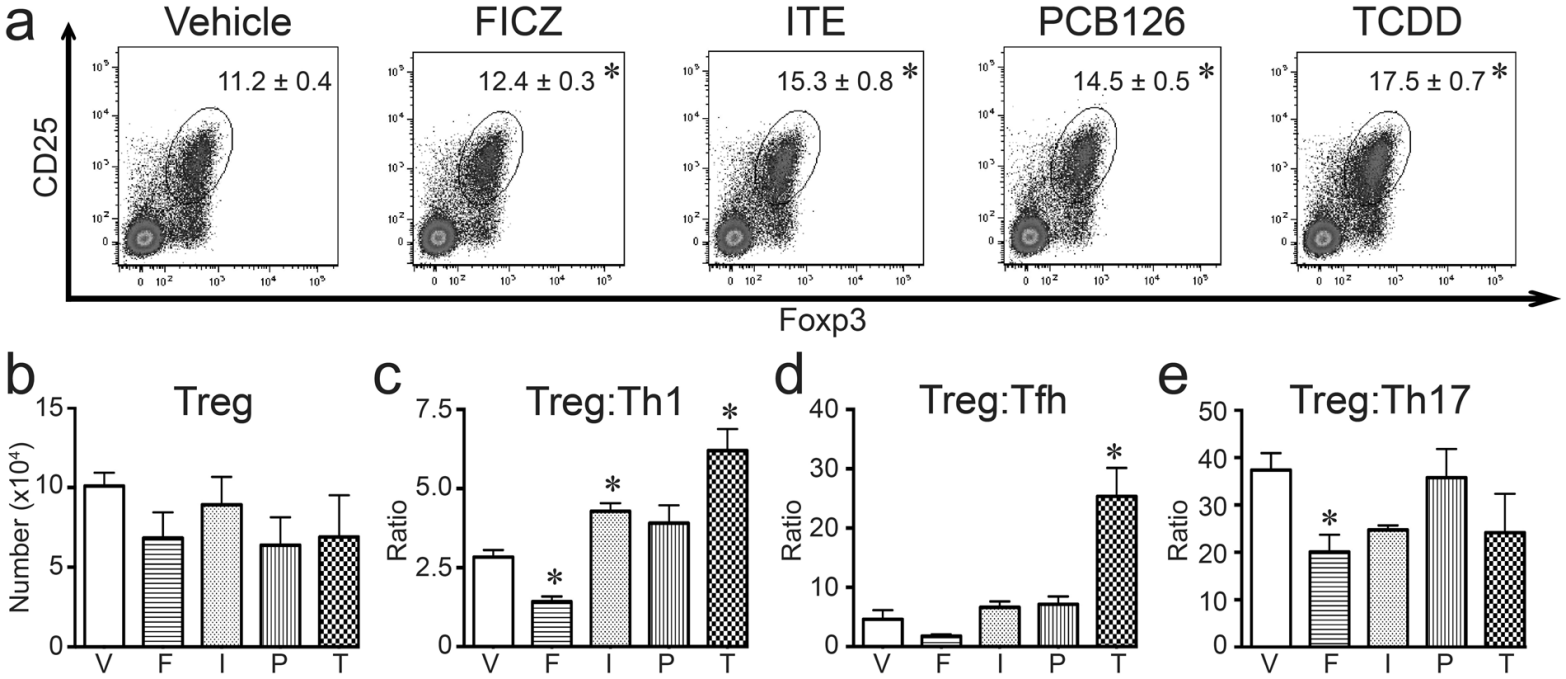
All four AHR ligands increase the frequency of Tregs during infection. Mice were exposed and infected as described in Fig. 1. MLN were harvested 9 days after infection, and cells were stained for flow cytometry. (**a**) Representative plots with mean percentage (± SEM) of Tregs (Foxp3^+^CD25^+^CD4^+^ cells; gated on CD4^+^ cells) are shown. (**b**) The graph shows the average number of Tregs in each group. (**c**–**e**) The graphs show ratio of (**c**) Treg:Th1 cells, (**d**) Treg:Tfh cells, and (**e**) Treg:Th17 cells. 5–8 mice were used per group, and an * indicates a p value ≤ 0.05 compared to vehicle control. Groups are designated as follows: V, vehicle control; F, FICZ; I, ITE; P, PCB126; T, TCDD. All experiments have been independently performed at least once, and the results were similar.

In addition to conventional CD4^+^ T cells, some AHR ligands change the proportion of regulatory CD4^+^ T cells (Tregs) in some, but not all, model systems^1,2,5,46,47^. Thus, we determined whether FICZ, ITE, PCB126, or TCDD affects Treg frequency in lung draining lymph nodes after IAV infection. All four compounds increased the percentage of Tregs (p ≤ 0.05; Fig. 4a), although the relative magnitude of this increase varied depending upon the ligand. There was no significant difference in the number of Tregs on day 9 post infection (Fig. 4b). The proportion of regulatory and conventional CD4^+^ T cell subsets provides a metric of a balanced response to infection. Compared to infected vehicle treated mice, the ratio of Treg:Th1, Treg:Tfh, and Treg:Th17 cells was generally reduced by exposure to FICZ (Fig. 4c,d). In contrast, ITE and TCDD enhanced the ratio of Tregs:Th1 and Treg:Tfh cells (Fig. 4c,d). Mice treated with PCB126 exhibited increased ratios of Tregs:Th1 and Treg:Tfh cells that mirrored changes with ITE, but differences were not statistically significant from control. The ratio of Treg:Th17 cells was not significantly affected by exposure to ITE, PCB126, or TCDD (Fig. 4e). Thus, overall, during IAV infection exposure to FICZ shifted the CD4^+^ T cell population toward more conventional versus regulatory CD4^+^ T cells, whereas ITE, PCB126, and TCDD shifted toward the opposite direction, with more Tregs compared to conventional CD4^+^ T cells.

### Differential effects of AHR ligands on T cell responses to IAV infection are tempered by dampening ligand metabolism

In the absence of CYP1A1-mediated breakdown, rapidly metabolized compounds, such as FICZ, are eliminated more slowly, which prolongs AHR activation^11,29,48,49^. Thus, using *Cyp1a1*^*−/−*^ mice in which metabolism of AHR ligands is slowed, we hypothesized that the effects of FICZ on T cell responses to IAV would be more similar to the effects of the other three compounds. TCDD significantly elevated expression of known AHR target genes *Ahrr* and *Cyp1b1* in liver by 26- to 50-fold in *Cyp1a1*^*−/−*^ mice (Table 1). PCB126 treatment resulted in a 6.4-fold increase in *Ahrr* expression and 17-fold increase in *Cyp1b1* expression, while ITE and FICZ elicited much more modest changes in these genes. These gene changes support that AHR signaling remains intact in *Cyp1a1*^*−/−*^ mice.

**Table 1:** Gene expression in Cyp1a1-deficient mice.

Gene	Ligand	Fold change	p-value
*Ahrr*	FICZ	1.29 ± 0.505	0.999
	ITE	1.86 ± 1.41	0.871
	PCB126	6.40 ± 2.58	0.740
	TCDD	26.37 ± 13.1	0.0068
*Cyp1b1*	FICZ	0.913 ± 0.248	0.902
	ITE	1.59 ± 1.01	0.894
	PCB126	17.8 ± 7.66	0.438
	TCDD	56.57 ± 29.2	0.0082

**Figure 5:**
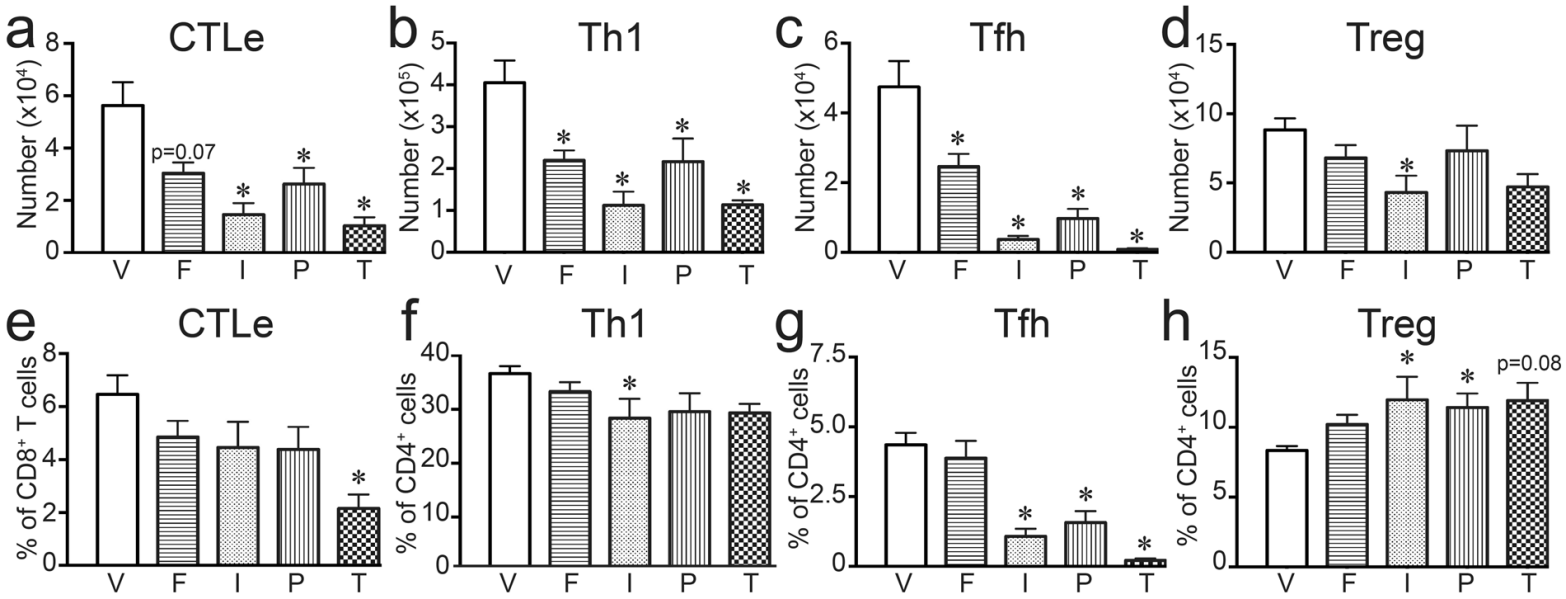
Reducing ligand metabolism results in all four compounds having similar effects. *Cyp1a1*^*−/−*^ mice (6–8 weeks of age) were treated with each compound or vehicle control, and infected with IAV using the same doses and timing outlined in Fig. 1. MLN cells were collected and stained for flow cytometry 7 days after infection. T cell populations were defined as in Figs 2–4. (**a**–**d**) The graphs depict the mean number (±SEM) of (**a**) CTLe, (**b**) Th1 cells, (**c**) Tfh cells, and (**d**) Tregs. (**e**,**f**) The graphs show the average percentage (±SEM) of (**e**) CTLe (of total CD8^+^ T cells) (**f**) Th1 cells (of total CD4^+^ T cells), (**g**) Tfh cells (of total CD4^+^ T cells), and (**h**) Tregs (of total CD4^+^ T cells). Groups are labeled as V, vehicle control; F, FICZ; I, ITE; P, PCB126; and T, TCDD. 4–8 mice were used per group, and an * indicates a p value ≤ 0.05 compared to the vehicle control group.

*Cyp1a1*^*−/−*^ mice. (6–8 weeks of age) were dosed and infected as in Fig. 5. Average *Ahrr* and *Cyp1b1* gene expression 7 days after infection, relative to infected mice treated with the vehicle control, was determined using RT-qPCR. Gene expression was measured in livers from 4–8 mice per group. The fold change indicates 2^−ΔΔCT^ ±SEM, and p-values were derived using an ANOVA, followed by Dunnett’s test.

Compared to vehicle treated *Cyp1a1*^*−/−*^ mice, treatment with all four compounds reduced the number of CTLe, although the FICZ-treated group did not reach p < 0.05 (Fig. 5a). However, all four compounds significantly reduced the number of Th1 and Tfh cells (Fig. 5b,c). Thus, the increased proportion of these CD4^+^ T cell subsets that was observed in FICZ treated wild-type mice was reversed in infected *Cyp1a1*^*−/−*^ mice (Fig. 5b,c,f,g). Also, FICZ treatment no longer elevated the percentage of Tregs in IAV infected *Cyp1a1*^*−/−*^ mice, although the percentage of Tregs remained significantly elevated by ITE and PCB126 treatment (p ≤ 0.05; Fig. 5h). A similar direction and magnitude effect on Tregs was observed in TCDD-treated *Cyp1a1*^*−/−*^ mice, but the difference from the vehicle-treated control was not statistically significant (p = 0.08; Fig. 5h). Collectively, these observations show that T cell responses during IAV infection are no longer differentially affected by FICZ versus the other three compounds when *Cyp1a1*^*−/−*^ mice were used. This suggests that reducing the metabolism of exogenously added ligands changes their effect on T cell responses.

### Hematopoietic AHR controls changes in adaptive immune responses to IAV

**Figure 6:**
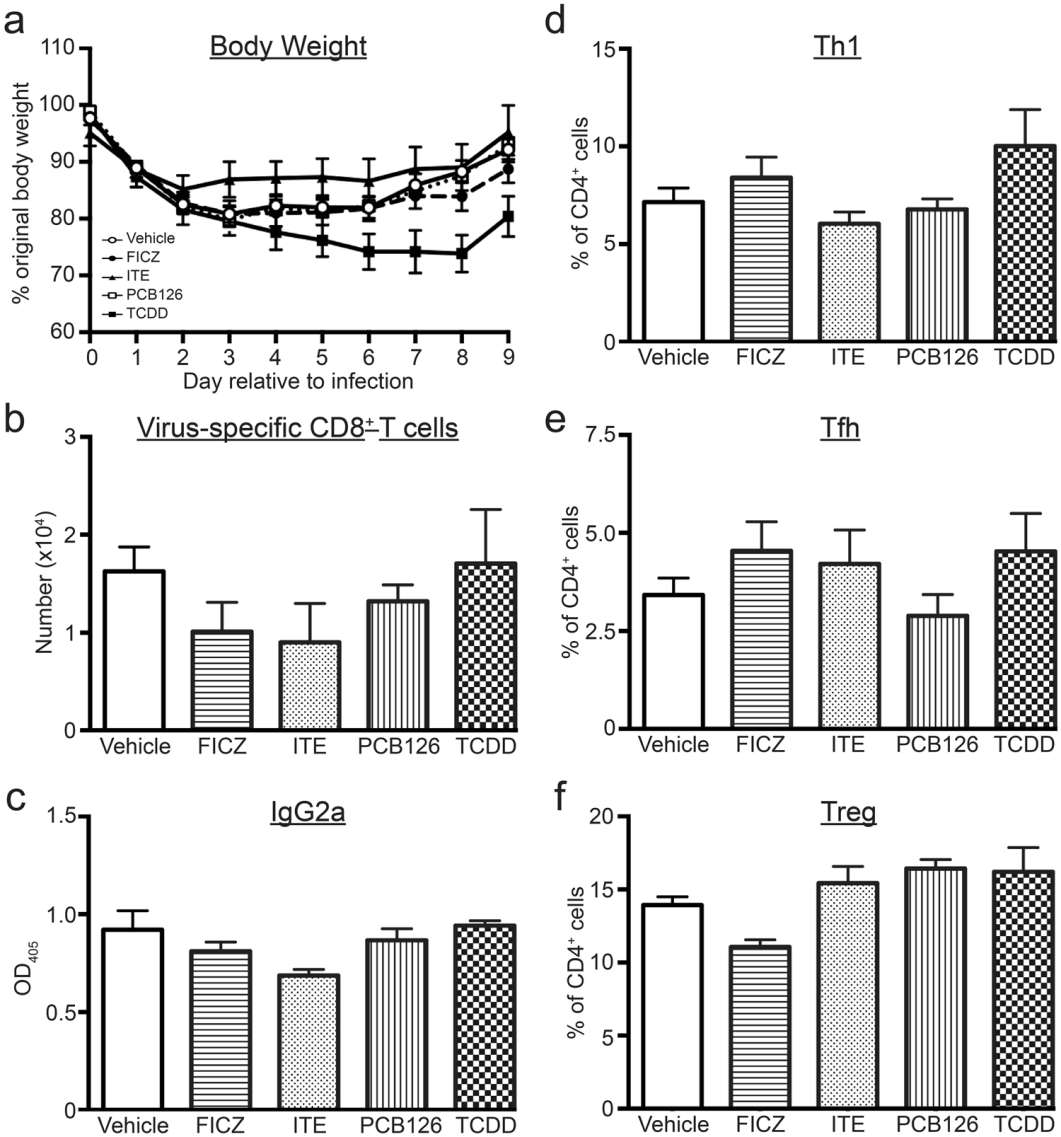
AHR mediates the effects of all four ligands on T cell and antibody responses to infection. Age-matched and sex-matched *Ahr*^*−/−*^ mice were treated with AHR ligands and infected with IAV, as in Fig. 1. MLN and serum were harvested on day 9 post infection, and MLN cells were stained for flow cytometry. T cell subsets were defined as in Figs 2–4, and virus-specific IgG levels were measured by ELISA. Graphs depict the (**a**) the percent body weight change relative to the day prior to infection for mice in all treatment groups, (**b**) number of virus-specific (D^b^NP_366–374_^+^) CD8^+^ T cells, (**c**) the relative amount of circulating anti-influenza IgG2a, (**d**) the percentage of TBet^+^CD4^+^ (Th1) cells, (**e**) the percentage of CD44^hi^CXCR5^+^PD-1^+^ CD4^+^ (Tfh) cells, and (**f**) percentage of Tregs (Foxp3^+^CD25^+^CD4^+^). 5–8 mice were used per treatment group. All data are shown ± SEM.

Both immune and non-immune cells express the AHR; however, it is not clear whether AHR-mediated changes triggered by each of these ligands arise due to AHR-driven events that are intrinsic or extrinsic to the immune system. Moreover, while a large body of evidence has established that most, perhaps all, actions of TCDD are mediated by the AHR, the extent to which the *in vivo* immunomodulatory effects of ITE, FICZ and PCB126 are AHR-dependent remains less well defined. As a prelude to conditionally ablating AHR from the immune system, we first sought to confirm that the *in vivo* modulation of adaptive immune responses to IAV infection caused by FICZ, ITE and PCB126 requires the AHR. Global *Ahr*^*−/−*^ mice were treated with each ligand and infected with IAV. Morbidity and the magnitude of antibody, CD8^+^ and CD4^+^ T cell responses after infection were measured. There were no statistically significant differences in body weight change after infection (Fig. 6a), and no mice died. Similar to prior reports using TCDD^43^, the number of virus-specific CD8^+^ T cells (Fig. 6b) and CTLe (data not shown) was not altered by exposure of *Ahr*^*−/−*^ mice to FICZ, ITE, or PCB126. Likewise, none of these compounds changed levels of circulating virus-specific IgG2a (Fig. 6c), the frequency of Th1 and Tfh CD4^+^ T cells(Fig. 6d,e), or Tregs (Fig. 6f) in MLN of *Ahr*^*−/−*^ mice. Thus, as with TCDD, the *in vivo* effects on the adaptive immune responses to IAV infection produced by FICZ, ITE, and PCB126 also require the AHR.

**Figure 7:**
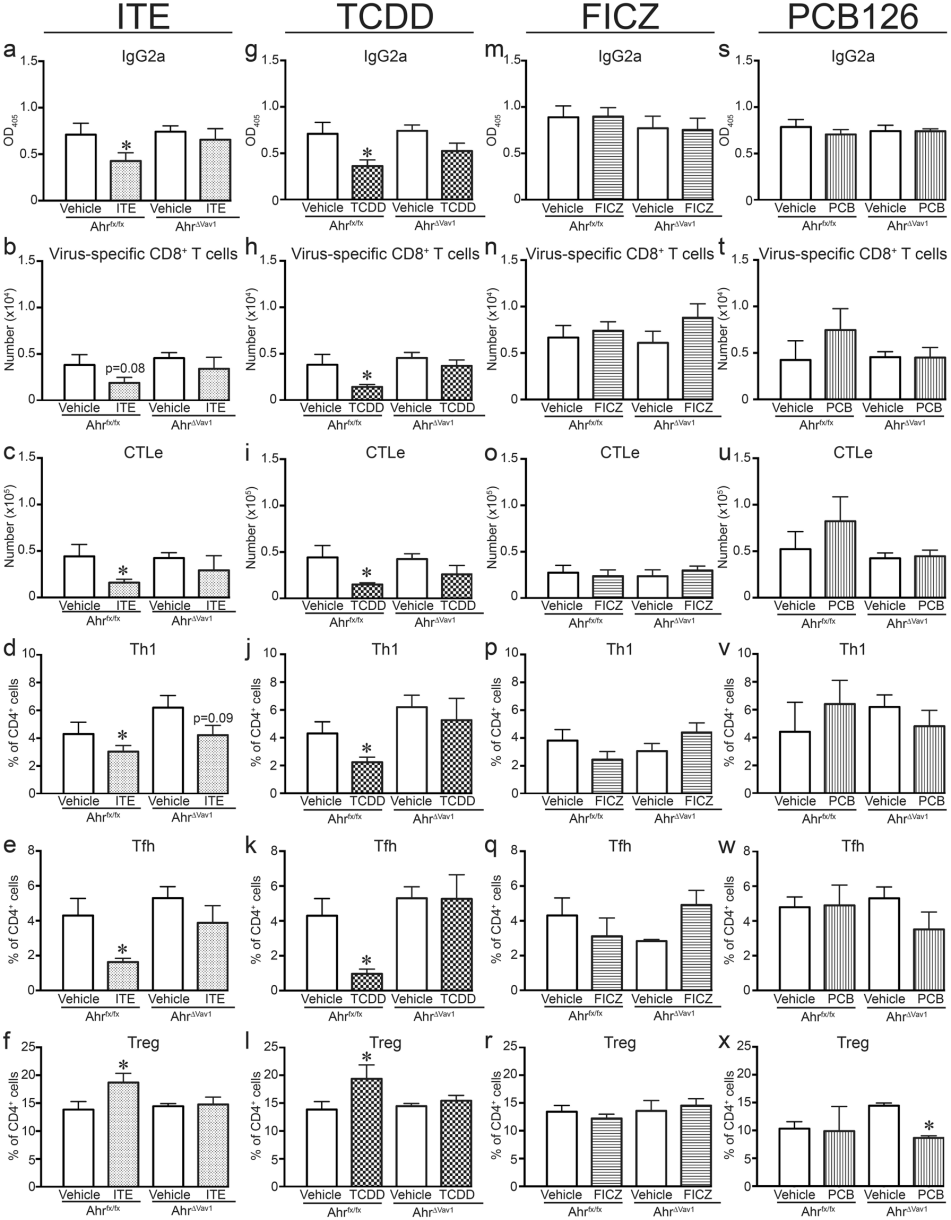
AHR in hematopoietic cells is necessary for changes in adaptive immune responses to IAV. *Ahr*^*ΔVav1*^ and *Ahr*^*fx/fx*^ mice were treated with AHR ligands and infected on day 0 as indicated in Fig. 1, except the doses were raised as follows: 1000 μg FICZ /kg BW, 1000 μg PCB126/kg BW, or 100 μg TCDD/kg BW. Daily treatment with ITE remained at 10 mg/kg BW. MLN and serum were collected 9 days after infection. CD4 and CD8 T cell subsets were defined using flow cytometry, and virus-specific antibody levels measured using ELISA. Row 1: Graphs **a**,**g**,**m**,**s** depict the relative level of circulating anti-influenza IgG_2a_. Row 2 (graphs **b**,**h**,**n**,**t**) shows the number of virus-specific (D^b^NP_366–374_^+^) CD8^+^ T cells, and Row 3 (graphs **c**,**i**,**o**,**u**) indicates the number of CTLe. Rows 4–6 show the percentage of Th1 cells (TBet^+^CD4^+^ T cells; graphs **d**,**j**,**p**,**v**), Tfh cells (CD44^hi^CXCR5^+^D-1^+^ CD4^+^ T cells; graphs **e**,**k**,**q**,**w**), and Tregs (Foxp3^+^CD25^+^CD4^+^; graphs **f**,**l**,**r**,**x**) in *Ahr*^*fxfx*^ and *Ahr*^*Vav1*^ mice treated with each compound. 5–8 mice per group were used for each experiment, and all data shown are ± SEM. An *indicates a p value ≤ 0.05 compared to vehicle control within each genotype.

To determine whether the observed changes in antibody, CD8^+^ and CD4^+^ T cell responses to IAV infection require AHR-mediated actions in hematopoietic cells, we conditionally ablated *Ahr* in the immune system. Specifically, *Vav1*^*cre*^ mice, which express Cre recombinase in all cells of hematopoietic origin^50^, were crossed with *Ahr*^*fx/fx*^ mice, thereby eliminating *Ahr* expression in all hematopoietic cells. We confirmed that *Vav1*^*cre*^ x *Ahr*^*fx/fx*^ (*Ahr*^*ΔVav1*^) mice do not express *Ahr* in cells isolated from the bone marrow, lymph nodes, or blood, but express *Ahr* in non-hematopoietic cells (data not shown). *Ahr*^*fx/fx*^ mice express the *Ahr*^*d*^ allele, which encodes an AHR protein with a 10-fold lower affinity for TCDD than *Ahr*^*b*^ alleles, including the b-1 *Ahr* allele expressed by C57Bl/6 mice^51,52^. Accordingly, the administered dose of FICZ, PCB126, and TCDD was increased 10-fold, although the dosing schedule and vehicle controls remain the same (i.e., as outlined in Fig. 1). The administered dose of ITE was not increased due to solubility, and also because the dosing strategy used in this study (10 mg/kg BW/d) has been previously shown to elicit immunological changes in *Ahr*^*d*^ mice^4^. Sex and age-matched *Ahr*^*Vav1*^ and *Ahr*^*fx/fx*^ mice were treated with each ligand (or respective vehicle controls) and infected with IAV. Figure 7 shows that the level of anti-viral antibodies, the number of virus-specific CD8^+^ T cells and CTLe, and frequency Th1 and Tfh cells in the MLN were significantly reduced in *Ahr*^*fx/fx*^ mice treated with ITE and TCDD (Fig. 7a–e,g–k). Similarly, TCDD and ITE increased the percentage of Tregs in lymph nodes compared to infected *Ahr*^*fx/fx*^ mice given vehicle control (Fig. 7f,l). Yet, none of these immunomodulatory effects of ITE or TCDD were observed in *Ahr*^*ΔVav1*^ mice (Fig. 7a–l. Yet, the 10-fold increase in the administered doses of FICZ and PCB126 were not sufficient to modulate the immune response to IAV infection in *Ahr*^*fx/fx*^ mice (Fig. 7m–x). As such, it is not possible to derive conclusive information regarding potential effects of these two compounds in these conditional knockout mice. Nonetheless, data from the *Ahr*^*Vav1*^ mice support that AHR activation within the hematopoietic system underlies the broad spectrum of changes in the adaptive immune response to IAV. Overall, this indicates that AHR within the hematopoietic system is an important target of AHR ligands, but that binding affinity for the AHR alone is not adequate to predict *in vivo* immunomodulatory consequences.

## Discussion

**Figure 8:**
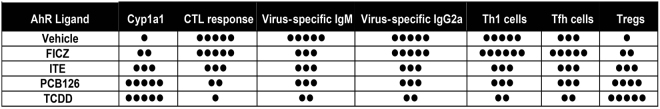
Summary of immune modulation during acute primary IAV infection. Exposure to each of these compounds modulates aspects of the adaptive immune response to IAV infection, although the magnitude, and sometimes direction of change, is ligand and cell type-specific. The number of dots represents the relative magnitude of the change in the indicated metric compared to infected vehicle-treated controls.

There is growing interest in manipulating the AHR to modulate the function of immune system to alleviate the progression of immune-mediated diseases and treat cancer. However, it has long been known that some AHR ligands, such TCDD, are profoundly immunosuppressive. This raises concern that pursuing the AHR as a novel therapeutic target carries a risk of unintended adverse consequences, such as poorer ability to fight infection. Also, although *in vitro* systems provide a means to screen activity and compare AHR ligands’ effects at a cellular level, few studies have directly compared different AHR ligands on the same *in vivo* immune response. This makes it difficult to predict how AHR binding compounds will affect *in vivo* host responses to infection. Moreover, when AHR ligands have been used *in vivo*, several reports describe a mixture of divergent and similar immunomodulatory effects, and we expand this knowledge base^1,2,5,11,47^. We show that *in vivo* treatment with each of these compounds affected aspects of the adaptive immune response to IAV infection (Fig. 8). Yet, differences in ligand metabolism and binding affinity influence the impact on specific lymphocyte subtypes and on aspects of the adaptive response to infection. This further supports that differences in the receptor, the ‘strength’ or duration of the signal, and events proximal to ligand-AHR interactions collectively influence the consequences of AHR activation on the immune response. This comparison provides important new information to consider as the AHR is explored as a potential therapeutic target and also for better understanding public health concerns about AHR-binding pollutants.

During IAV infection, AHR activation influenced Tregs, Th1, Tfh cells, but not Th17 cells. This further reinforces the idea that AHR signaling affects CD4^+^ T cells in a T cell subset- and ligand-specific manner, but also illustrates that the type of antigenic challenge plays an important role in determining which CD4^+^ T cell subsets are sensitive to AHR activation. For example, studies using mouse models of other diseases have shown ligand-specific alterations in conventional and regulatory CD4^+^ T cell populations^1,2,47,53,54^. For instance, after induction of experimental autoimmune encephalomyelitis (EAE), the frequencies of Th1 and Th17 cells are increased by FICZ, but decreased by ITE or TCDD treatment. Yet, ITE and TCDD, but not FICZ, augmented Tregs in EAE^1,2,4^. Similarly, mesalamine activates AHR, and enhances Tregs in the absence of modulation of Th1 and Th17 cells during inflammatory bowel disease, while treatment with 10-chloro-7H-benzimidazo[2,1-a]benzo[de]iso-quinolin-7-one (10-Cl-BBQ) diminishes Th17 cells but not Th1 cells in NOD mice^5,10^. Thus, the absence of an effect of the four AHR ligands on Th17 cells in this present study may reflect that they are not the principal conventional CD4^+^ T cell type active during mild IAV infection. In essence, AHR activation may preferentially affect particular CD4^+^ T cell subsets during the immune response to a specific antigen. This may also reflect that AHR signaling in accessory cells has a strong influence on CD4^+^ T cells. In support of this idea, AHR activation in dendritic cells (DCs) changes their function and contributes to altered T cell responses^4,55,56^. Thus, the particular conventional CD4^+^ T cell subsets affected could be due to direct, AHR-mediated changed in T cells themselves, and also from AHR-driven changes in immune cells that interact with T cells during the course of an immune response, including DCs.

In addition to skewing differentiation of Th1 and Th17 conventional CD4^+^ T cell subsets, which has been previously reported, we demonstrate that AHR activation changes the proportion of Tfh cells during IAV infection. Tfh cells are critical for proper B cell activation, antibody isotype switching, affinity maturation, and memory cell formation^57^. While AHR signaling has not previously been associated with effects on Tfh cells, given the impact of AHR ligands on T cell dependent antibody responses^58–60^, it is logical that Tfh cells are sensitive to modulation by AHR signaling. Reduced Tfh cell help to B cells leads to a poorer class-switched virus specific antibody response^61^. Consistent with this, we observed an association between exposure to agents that reduced Tfh cells and dampened virus-specific IgG levels during IAV infection. This suggests that reductions in the frequency of Tfh cells may contribute to reduced levels of circulating antibodies after vaccination, which have been reported in human populations exposed to pollutants that activate the AHR, including PCBs and dioxins^62–64^. On the other hand, *in vivo* treatment with FICZ elevated Tfh cells during IAV infection. Whether there are consequences of this increase in Tfh cells on antibody levels remains uncertain. During primary IAV infection, further elevating Tfh cell number did not promote increased virus-specific IgG levels 9 days after infection. However, the long-term consequences of a larger pool of Tfh cells on virus-specific antibody levels remain unexplored. It is possible that boosting the number of Tfh cells during infection leads to a heightened effect on the overall trajectory of isotype-switched antibody. If so, then the consequences of more Tfh cells on the antibody response may not be apparent until later on.

When placed into a broader perspective, these collective results illustrate the importance of integrating assessments of the effects of AHR ligands across multiple metrics of immune function. For instance, although there were divergent effects on virus-specific IgG levels and T cells, all four compounds significantly dampened virus-specific IgM levels. IgM is released by B cells via a CD4^+^ T cell-independent pathway. This is consistent with prior reports showing that TCDD and ITE modulate B cells independently of T cells^65,66^. Thus, similar to other AHR binding compounds, FICZ and PCB126 may also directly affect B cells, leading to reduced IgM production. *Cyp1a1* induction provides another example that emphasizes the importance of integrated assessments. Although treatment with FICZ changed several aspects of the immune response to infection, relative to the other three AHR ligands used, the fold induction of *Cyp1a1* was much less. This may reflect the rapid metabolism of FICZ, but also suggests a more generalizable concept: *Cyp1a1* is not a reliable marker for an AHR ligand’s effects on immune system. This is not to say that induction of *Cyp1a1* should be disregarded; indeed, a modest induction of *Cyp1a1* in gene expression profiling sparked the discovery that the anti-inflammatory drug mesalamine ameliorates inflammatory bowel disease by interacting with the AHR^10^. Nonetheless, the findings presented here further support the emerging idea that *Cyp1a1* is not an ideal reporter of immunomodulatory AHR activity. Precisely which target genes provide definitive indicators of AHR-mediated immune effects remain elusive, and the target genes may depend upon cell type, microenvironment, and disease context. Thus, when examined in isolation, the magnitude of the increase in AHR-driven *Cyp1a1*/CYP1A1 is not a dependable hallmark of immune modulation. Although it indicates AHR activation, it does not provide reliable or predictive information regarding the direction or magnitude of immune responses. Consequently, measurements of *Cyp1a1*/CYP1A1 should be combined with *in vivo* measurements of immune function. This integration is important to ensure that therapeutic agents are not discarded prematurely, and that potential immunotoxicity is not overlooked because a compound of interest causes only modest induction of *Cyp1a1*/CYP1A1.

Extending these ideas further, the findings reported herein suggest that TCDD does not represent a unique or peculiar AHR ligand. Prolonging exposure to rapidly degraded ligands, such as FICZ, by reducing their metabolism, lessened the responses of conventional helper CD4^+^ T cell and CTL during IAV infection. This is consistent with other reports, and is likely due to the rapid *in vivo* metabolism of FICZ. For instance, *in vitro* treatment with FICZ and ITE induce gene expression changes similar to those induced by TCDD, with the main differences relating to timing and concentration^25,67^. Thus, the immune modulation caused by TCDD may be indicative of the potential actions or pathways affected by other AHR ligands, with the main difference being that TCDD’s chemical structure renders it refractory to metabolism and elimination.

Another important, yet unexpected, finding arose through use of the cre-loxP system. Several groups have reported that different tissue and cell types are likely sensitive to AHR activation, including liver, endothelial cells, respiratory epithelial cells and leukocyte lineages^52,55,56,68–70^. However, there is limited comparative information regarding how hematopoietic-specific AHR signaling *in vivo* impacts immune modulation by different ligands. Consistent with prior reports, the work reported here shows that global *Ahr* deletion attenuates AHR-mediated changes to adaptive responses during acute infection, and that modulation of the adaptive immune response to IAV infection requires AHR expression in the hematopoietic system^10,43,46,68,71^. What is surprising is that, even using a 10-fold higher dose than was used in C57Bl/6 mice, neither PCB126 nor FICZ modulate the immune response to IAV infection in *Ahr*^*fxfx*^ mice. *Ahr*^*fx/fx*^ mice express a functional AHR protein. A key difference between *Ahr*^*fx/fx*^ mice and wild-type C57Bl/6 mice is the *Ahr* allele expressed: *Ahr*^*fx/fx*^ mice are *Ahr*^*d*^ whereas wildtype express the *Ahr*^*b*^ allele. Based primarily on studies with TCDD, there is a 10-fold difference in binding affinity between *Ahr*^*b*^ and *Ahr*^*d*^ alleles (b > d; ref.^51^). Our results suggest that the fold-change in binding affinity may not follow the same ‘scaling factor’ for all AHR ligands. In other words, even though the affinity for TCDD is 10-fold different between mouse *Ahr*^*b*^ and *Ahr*^*d*^ alleles, it may not be valid to assume other ligands follow this same 10-fold difference in affinity. Structure-activity studies are needed to define the affinity of emerging AHR ligands for different alleles, because they may not change following the same proportion as TCDD. Consequently, dose-response studies with each particular ligand will need to be performed to define the tissue and cell-type specific consequences of *in vivo* AHR activation in future studies.

In addition to differences in metabolism and binding affinity, different chemicals may influence the interactions of the AHR with cofactor proteins that influence intracellular signaling pathways^22,72^. This is a common feature of nuclear receptors, and contributes to modifications to cellular events downstream of their engagement^73,74^. For example, the AHR has been shown to interact with subunits of the nuclear factor kappa b (NF-κB) complex in myeloid cells, B cells, and fibroblasts^75–77^, although the precise NF-κB family members with which AHR interacts varied among cell types. Thus, interactions with cofactors are likely cell-type specific, which may help to explain some of the cell-type specific effects observed among the AHR ligands. Yet, AHR cofactors within immune cells are not extensively explored. Moreover, even in carefully defined, homogeneous *in vitro* systems, different ligands can elicit distinct responses^31,78,79^. Thus, differences in a complex *in vivo* immune response cannot be simply attributed to one dimension, such as cell-type specific factors or ligand metabolism. The complex interplay between characteristics of the ligand and cell type contributes to the consequences of AHR engagement.

In summary, through the work reported herein, we have expanded knowledge regarding how different AHR agonists influence adaptive immune responses. Regardless of which ligand was used, AHR activation modified the immune response to IAV infection, but the consequences varied in a ligand-specific manner, conceivably due to differences in the quality, magnitude, and/or duration of AHR signaling. When considering the AHR as a therapeutic target, the net influence likely reflects interactions between the particular immune cells involved in responding to antigen challenge, dosage, the biochemical properties of the AHR ligand used (metabolism, binding affinity), and potentially the *Ahr* allele expressed. Furthermore, the ‘directional’ differences among changes wrought by FICZ vs. other agents suggest that AHR activation leads to simultaneous but independent events within multiple cell types, which collectively influence antiviral immunity. Although this work was conducted in the context of IAV infection, adaptive immune responses are central to fighting other communicable diseases, and also contribute to numerous disease states. Thus, the implications translate to improved ability to predict the potential effects of the diverse range of AHR ligands on host defenses against not only IAV, but other infections and, perhaps also, adaptive immunity toward non-infectious challenges.

## Methods

### Animals and treatment

C57Bl/6 mice (age 5–6 weeks) were purchased from the Jackson Laboratory or the National Cancer Institute Mouse Repository. Initial breeding stocks for B6.AhR^tm1Bra^ (*Ahr*^*−/−*^) and *Ahr*^*fx/fx*^ mice^52,80^ were provided by Dr. Christopher Bradfield (University of Wisconsin), *Vav1*^*cre*^*Ahr*^*fx/fx*^ (*Ahr*^*ΔVav1*^) breeders were from Dr. Thomas Gasiewicz (University of Rochester), and initial stock for *Cyp1a1*-deficient (*Cyp1a1*^*−/−*^) mice^81^ was provided by Dr. Daniel Nebert (University of Cincinnati). All data presented are from female mice that were 6–12 weeks of age at the time of experiments. Some experiments were also conducted using male mice, which demonstrated similar effects of AHR activation on the immune response to IAV (data not shown). All mice were housed in microisolator cages in a specific-pathogen free facility at the University of Rochester, and were provided food and water ad libitum. All animal treatment was conducted with prior approval of the Institutional Animal Care and Use Committee of the University of Rochester, and following all guidelines and regulations.

2,3,7,8-Tetrachlorodibenzo-p-dioxin (TCDD; Cambridge Isotopes, Tewksbury, MA) and 3,3′,4,4′,5-pentachlorobiphenyl (PCB216; AccuStandard Inc., New Haven, CT) were dissolved in anisole and diluted in peanut oil. 2-(1*H*-Indol-3-ylcarbonyl)-4-thiazolcarboxylic acid methyl ester (ITE; Tocris Bioscience, Minneapolis, MN) was dissolved in acetone and diluted in peanut oil. 6-Formylindolo[3,2-b]carbazole (FICZ; Enzo Life Sciences, Farmingdale, NY) was dissolved in DMSO and diluted in peanut oil. C57Bl/6 (*Ahr*^*b*^) mice were treated with 10 μg TCDD/kg body weight (BW), 100 μg PCB126/kg BW, or 100 μg FICZ/kg BW by gavage. Mice were treated with 10 mg ITE/kg BW by i.p. injection. Dosing schedules are shown in Fig. 1a. Due to the different solubilization conditions, separate groups of mice received the appropriate vehicle control on the same dosing schedule and via the same route that the compound was administered. Thus, one group of vehicle-exposed mice was treated once by gavage (VEH_DLC_), and served as the control group for TCDD and PCB (dioxin-like compounds, or DLC). Other mice were gavaged daily as the control for FICZ treatment (VEH_FICZ_), and another group was treated i.p. daily to provide controls for ITE (VEH_ITE_). No differences were observed in any metrics of the immune response to IAV infection among the 3 vehicle control groups (data not shown); therefore, aggregate vehicle group (VEH) data are presented in the figures. For experiments with *Ahr*^*fx/fx*^ or *Ahr*^*ΔVav1*^ mice (*Ahr*^*d*^), ligands were administered via the same route, but the doses were changed to 100 μg TCDD/kg BW, 1000 μg PCB126/kg BW, and 1000 μg FICZ/kg BW to compensate for ligand binding affinity differences between the *Ahr*^*b*^ *and Ahr*^*d*^ alleles^82^.

### Infection

Influenza A virus (IAV) strains A/Memphis/102/72 (Mem/102; H3N2) or A/HKx31 (HKx31; H3N2) were prepared, titered, and stored as previously described^37^. Mice were anesthetized by intraperitoneal (i.p.) injection of avertin (2,2,2-tribromoethanol; Sigma Aldrich, St. Louis, MO) and infected intranasally (i.n.) with 120 hemagglutinating units (HAU) of HKx31 or 1 × 10^7^ plaque forming units (PFU) Mem/102 diluted in endotoxin tested PBS. These viral inocula do not cause mortality in immunocompetent mice. All work with infectious agents was conducted with prior approval of the Institutional Biosafety Committee of the University of Rochester, following guidelines of the NIH/CDC.

### Tissue collection

Blood was collected by cardiac puncture, and serum was stored at −80 °C. Immune cells were isolated from the mediastinal lymph nodes (MLN) and lung as previously described^56,83^. After hypotonic lysis to remove erythrocytes, single cell suspensions were prepared. CD45^negative^ (non-hematopoietic cells) were isolated from the lung using protease digestion^84^. Briefly, 1% low melting point agarose was inserted into the lung via the trachea, and the tissue digested with dispase (Stem Cell Technologies, Cambridge, MA). CD45^negative^ cells were isolated from the resulting cell suspension using magnetic beads conjugated to an anti-mouse CD45 antibody (Miltenyi Biotec, Auburn, CA).

### PCR and reverse transcription(RT)-qPCR

Genotyping of *Ahr*^*−/−*^, *Ahr*^*fx/fx*^, *Ahr*^*ΔVav1*^ and *Cyp1a1*^*−/−*^ mice was performed using PCR, as previously described^11,52,56,69,80,81^. To verify *Ahr* excision in *Ahr*^*ΔVav1*^ mice, genomic DNA was isolated from CD45^+^ and CD45^negative^ cells using a QIAamp DNA Mini Kit (QIAGEN, Valencia, CA), and PCR was performed to detect the excised *Ahr* allele, as previously described^56^. Tissues were removed and immediately snap frozen in liquid N_2_. For real time-RT-PCR (RT-qPCR) to measure gene expression, RNA was isolated from lung or liver using the QIAGEN RNeasy Mini Kit. RNA was reverse transcribed, and the following gene-specific primers used: *Ahrr* (5′GAGGCCAGGTCCCAGAGATGAGAGA3′; 5′GGGGCGCAGAAGATCGGGCG3′; IDT), *Cyp1a1* (5′TTTGGAGCTGGGTTTGACAC3′; 5′CTGCCAATCACTGTGTCTA3′; IDT)^56^, and *Cyp1b1* (5′CTAAGGTCCCGCTTCCTCCA3′; 5′ATGCGCCATCCCTCTACTCC3′; IDT). RT-qPCR was performed using a Bio-Rad iCycler MyiQ2 with IQ SYBR Green Supermix (Bio-Rad). *L13* was used as an internal control gene (5′CTACAGTGAGATACCACACCAAG3′; 5′TGGACTTGTTTCGCCTCCTC3′, IDT). Gene expression in each ligand-treated group was compared to the vehicle control group using the 2^−ΔΔC^_T_ method^85^.

### Flow cytometry

Isolated cells were incubated with previously determined optimal concentrations of the following fluorochrome-conjugated monoclonal antibodies: CD3ε (PE-Cy5; clone 145–211), CD4 (PerCP-Cy5.5; clone RM4–5), CD8α (APC-Cy7; clone 53-6-7), CD25 (APC: clone PC61.5), CD44 (eF450: clone IM7), CD62L (PE-Cy7; clone MEL-14), CXCR5 (Biotin, with PE-conjugated streptavidin: clone 2G8), and PD-1 (FITC; clone J43)^44^. IAV-specific CD8^+^ T cells were identified using allophycocyanin (APC)-labeled major histocompatibility (MHC) class I tetramers containing an immunodominant viral epitope of HKx31 (nucleoprotein, D^b^NP_366–375_)^43^. To identify CD4^+^ T cell subsets, cells were incubated with fluorochrome-conjugated antibodies against CD4 and CD25, fixed and permeabilized using the Foxp3 Staining Kit (eBioscience, San Diego, CA), and then incubated with fluorochrome-conjugated antibodies against Foxp3 (AF700; clone FJK-16S), GATA3 (AF488; clone L50-823), RORγt (PE; clone Q31-378), and TBet (PE-Cy7: clone 4BIO)^44,86^. Non-specific staining was blocked by incubating cells with an anti-mouse CD16/32 mAb. All antibodies were purchased from eBioscience (San Diego, CA) or BD Biosciences (San Jose, CA). Fluorescence minus one (FMO) controls were used to determine non-specific fluorescence and define gating parameters. Data were collected using an LSRII flow cytometer (BD Biosciences, San Jose, CA), and analyzed using the FlowJo software program (TreeStar, Ashland, OR).

### Anti-influenza virus antibody ELISA

Relative influenza virus-specific antibody levels in serum were measured by antibody isotype-specific enzyme linked immunosorbent assays (ELISA) as previously described^87^. Briefly, 96-well plates were coated with density gradient purified IAV (Charles River, Wilmington, MA), and after blocking to reduce non-specific binding, serum samples were serially-diluted. Biotinylated anti-isotype-specific antibodies (Southern Biotech, Birmingham, AL) were added, following by avidin-peroxidase. 2,2′-azino-bis(3-ethylbenzothiazoline-6-sulphonic acid) substrate was used to induce colorimetric change. Absorbance values were read at 405 nm using a SpectraMax Plate reader (Molecular Devices, Sunnyvale, CA).

### Virus titer assay

The pulmonary viral burden was measured by determining the number of foci forming units (FFU) in lung homogenates^88^. Briefly, lungs were harvested from mice and mechanically homogenized. Serially diluted lung homogenates were incubated overnight on confluent Madin-Darby canine kidney (MDCK) cells in 96 well plates. The next day, plates were incubated with biotin-conjugated influenza A nucleoprotein specific antibodies (Millipore, Billerica, MA), followed by streptavidin-conjugated alkaline peroxidase (Southern Biotech, Birmingham, AL), and BCIP-NBT as the colorimetric substrate (Sigma-Aldrich, St. Louis, MO). Foci counts were adjusted to the dilution of lung homogenate, and the data are expressed as FFU/lung.

### Statistical analysis

Data were analyzed using JMP software (SAS, Cary, NC). Differences between groups were evaluated using a one-way analysis of variance, with a Dunnett’s post-hoc test, using the appropriate vehicle control as the control group. A Mann-Whitney U test was used to analyze changes in body weight over time. Differences were considered significant when p values were less than or equal to 0.05. Error bars on all graphs represent the standard error of the mean (SEM). Experiments were performed with at least 5 age- and sex-matched mice per treatment group at each point in time.

### Data availability

Data generated and analyzed during this study are available from the corresponding author upon reasonable request.
